# Error and bias in race and ethnicity descriptions in medical examiner records in New Mexico: Consequences for understanding mortality among Hispanic/Latinos

**DOI:** 10.1016/j.fsisyn.2023.100338

**Published:** 2023-06-08

**Authors:** Nicollette S. Appel, Heather J.H. Edgar, Shamsi Daneshvari Berry, Keith Hunley

**Affiliations:** a Department of Anthropology, University of New Mexico, USA; b Office of the Medical Investigator, University of New Mexico, USA; c Department of Biomedical Informatics, Western Michigan Homer Stryker MD School of Medicine, USA; d Department of Pathology, University of New Mexico, USA

**Keywords:** Medicolegal death investigation, Race, Ethnicity, Structural vulnerability, Cognitive bias

## Abstract

Researchers use public records from deceased individuals to identify trends in manners and causes of death. Errors in the description of race and ethnicity can affect the inferences researchers draw, adversely impacting public health policies designed to eliminate health inequity. Using the New Mexico Decedent Image Database, we examine: 1) the accuracy of death investigator descriptions of race and ethnicity by comparing their reports to those from next of kin (NOK), 2) the impact of decedent age and sex on disagreement between death investigators and NOK, and 3) the relationship between investigators’ descriptions of decedent race and ethnicity and cause and manner of death from forensic pathologists (n = 1813). Results demonstrate that investigators frequently describe race and ethnicity incorrectly for Hispanic/Latino decedents, especially regarding homicide manner of death and injury and substance abuse causes of death. Inaccuracies may cause biased misperceptions of violence within specific communities and affect investigative processes.

## Highlights

• Hispanic/Latino decedents were underreported by death investigators.
• Decedents are overreported as White in cases of homicide.
• Cause of death by substance abuse or injury were related to misidentification.
• There is systematic bias in investigators' assignment of race and ethnicity.

## 1 Introduction

### 1.1 Overview

Vital records from deceased individuals are used by public health researchers to identify trends in causes and manners of death. This research informs public policies designed to eliminate disparities in health that are related to sex, race, and ethnicity [[1], [2], [3], [4]]. Research dating as far back as the 1960s has shown that there are often errors in these records related to the determination of decedent race and ethnicity, as well as manner and cause of death (reviewed in Ref. [5]). At the individual level, such errors may affect investigations; at the population level, they may adversely affect public policies aimed at eliminating racial and ethnic disparities associated with specific causes and manners of death.

Within the medicolegal system, forensic anthropologists have unique expertise regarding the ways racial disparities and marginalization are embedded within methodologies and practices. Conversations about the situationality of medicolegal investigation in recapitulating inequity are becoming central in forensic anthropology. The inclusion of a structural vulnerability profile is a logical next addition to forensic anthropology casework. This kind of profile considers the social inequities experienced by a living individual. These disparities become embodied biologically and can be documented in decedent remains [6,7]. However, the results of forensic casework and the public health research that derives from it are only as good as the data that comprise it.

The information in public records about deceased people are drawn primarily from law enforcement records, crime laboratories, death certificates, and medical examiner records. Here, we address the errors that originate in medical examiner's records, which sometimes propagate to death certificates. Further, we identify the rate at which misidentifications are made, and which errors are most common. Age, sex, manner of death, and cause of death classifications were analyzed to recognize any bias in race and ethnicity identifications. The results of this study are considered in the context of applying a structural vulnerability framework to medico-legal casework, including but not limited to forensic anthropological analyses.

### 1.2 Structural vulnerability profile

The purpose of this special journal issue is to “conceptualize social vulnerability assessments and structural violence data in forensic casework.” A structural vulnerability profile (SVP) includes information on the person's race, ethnicity, socioeconomic status, and health status, and considers how this information can be used as part of the forensic investigative process. The demographic parts of this profile are described and documented on death certificates, and the lived experience aspects can be used to interpret the cause and manner of death. Public health sectors value the demographic information included on death certificates to examine associations with cause and manner of death. Aggregated, decedent's demographic information clarifies public health problems within the larger general population. Inaccuracies constrain the effectiveness of public health changes, limiting their positive impact on minority populations. The CDC's instructions for filling out death certificates regarding Hispanic origin and race state that the information included will not appear on any certified copy of the death certificate. Even if it does not appear on death certificates, data about Hispanic origin, “is needed to identify health problems in a large minority population in the United States. Identifying health problems will make it possible to target public health resources to an important segment of our population.” (Items 52 on the U.S. Standard Certificate of Death, CDC, 2003). Similarly, race “is essential for identifying specific mortality patterns and leading causes of death among different racial groups. It is also used to determine if specific health programs are needed in particular areas and to make population estimates.” (Items 53 on the U.S. Standard Certificate of Death, CDC, 2003). Note that death certificates in New Mexico include information on race, ethnicity (Hispanic/Latino or not), and Tribal affiliation, contra CDC instructions. In the context of incorporating an SVP into investigations of death, the findings of this study will show how inaccuracies in data from medical examiner records broadly impacts vital records, research using medical examiner data, and public health directives.

The value of this analysis comes from the need to better understand the populations affected by structural vulnerability. Before the work can be done to incorporate a structural vulnerability profile to forensic casework in any capacity, the first requirement is to recognize those populations most at risk of being affected by marginalization and structured violence. Only then can the patterns of structural vulnerability be identified and applied. In the context described by Ref. [6] there is an assumption that the ways lived social inequities can affect specific groups is known. This kind of information derives from research spanning the fields of public health and clinical medicine to medical anthropology and bioarchaeology (Caruth et al.*,* 2021 [7,8]; Klaus, 2012). The identification of the patterned errors and bias in the assignment of race and ethnicity at the very least brings attention to complication inherent to the current medico-legal system. The larger goal is to ensure that an SVP is used appropriately and effectively. If this research draws on medical examiner records, death certificates, or vital records, and errors exist due to systemic biases in the medico-legal setting, then analyses on population vulnerability will have inaccuracies.

### 1.3 Death certificates

Although they vary from state to state, in general, death certificates list: 1) demographic data such as age, sex, race, and ethnicity; 2) information surrounding the death, such as the date, time, and manner and cause of the death; and 3) information about disposition of the body [9,10]. The CDC's National Center for Health Statistics (NCHS) has created a standard death certificate form, but states are allowed to vary from this. The CDC also sets guidelines on the registration of death for funeral directors, physicians, and medical examiners/coroners, as these are the three main groups of individuals who can certify death certificates. Additionally, NCHS offers training on how to collect, code, edit, and transmit race and Hispanic-origin data, as well as how to code and classify causes of death.

Despite these efforts to standardize death certificates and train certifiers, errors on death certificates are common [11]. An examination of 494 death certificates from Baltimore, Maryland between the years of 1995 and 1997 found that the cause of death was improperly recorded 41% of the time [12]. Similarly, a retrospective study that looked at 601 death certificates in Vermont found that 51% had errors that were classified as major errors that might affect interpretations of manner and causes of death [13]. Errors included recording an incorrect cause of death and manner of death. These errors potentially impact our understanding of racial and ethnic differences in health. For example [14], compared racial classification on 22,905 death certificates to that provided by NOK [15]. While the overall level of disagreement was low at 1.1%, it was racially patterned, with low disagreement for white individuals at 1% and high disagreement for American Indian individuals at 8.8%. Using the same dataset [16], quantified misclassification of Hispanic Americans on death certificates and demonstrated that the misclassification biased Hispanic mortality rate downward and falsely inflated life expectancy.

### 1.4 Medical examiner records

Medical examiner records contain more in-depth information about decedents than do death certificates, and include data derived from death investigators. These records document portions of police reports, the autopsy and postmortem examination, toxicology results, and reports from consultants such as forensic anthropologists and odontologists [17]. These additional sources of information create a more complete picture of the circumstances surrounding death than does a death certificate alone. However, errors in medical examiner data also appear common in some states. Comparing violent injury death reporting by the Medical Examiner and the Vital Statistics Office in Oklahoma [18], concluded that the Medical Examiner system over-reported homicides. In a study of firearm deaths [19], compared medical examiner data and vital records data in Multnomah County, Oregon from 2010 through 2016. The author found that the two sources agreed for sex, age, and manner of death, but they differed substantially in the characterization of race and ethnicity, particularly for Hispanic ethnicity (sensitivity = 62%). While these studies demonstrate that reporting errors are common, additional studies are required to identify the nature and magnitude of the problem in different parts of the US, where distinctive social identities and health inequities have developed in response to unique social and historical conditions.

### 1.5 Death investigations in New Mexico

New Mexico is an ideal location for this study as New Mexico has a centralized medical examiner system. This means there is one central office, the Office of the Medical Investigator (OMI), that manages death investigations for the state. The OMI is staffed by medical examiners; there are no elected coroners. OMI is required to investigate “any death that occurs suddenly and unexpectedly when the person has not been under medical care, suspected to be due to violence, alcohol, intoxication, or exposure to toxic agents, in nursing homes or other private institutions without recent medical attendance, deaths unattended by a physician, and occurring under suspicious circumstances” [20]. Further, an autopsy is required when investigators suspect the death was caused by a criminal act or when the cause of death is obscure. Autopsies are performed by medical examiners who are qualified forensic pathologists, certified by a state board, ensuring that cause and manner of death are determined by highly skilled professionals.

Within the OMI system, there are two employment classes of death investigators. Central office investigators work full-time at the OMI main office and serve the state's main population center, Albuquerque, in Bernalillo County. Field investigators work part-time in the more rural counties of the state. Many have backgrounds in law enforcement, as first responders, or funeral directors. Race and ethnicity are recorded by these death investigators who are often familiar with the local demographics of the counties they serve, which in principle should lead to accurate description of decedent race and ethnicity.

For OMI cases with an identified NOK, death certificate demographic information is likely to come from NOK. However, in cases of indigent or unclaimed individuals, funeral homes rely on demographic information provided by the OMI. This information derives from the death investigation, through documents, driver's licenses, and correspondence with neighbors and friends (Tovar, personal communication, 2022). Investigators may also rely on their own estimation of demographic information about decedents, including sex, race, and ethnicity. In these circumstances, the information documented within the medical examiner records is then included on death certificates. Any errors in the initial records are maintained in the death certificate and becomes part of the vital records for the state.

New Mexico has a dataset that is well-suited for this study, the New Mexico Decedent Image Database (NMDID), which contains whole-body postmortem Computed Tomography (CT) scans of thousands of New Mexico decedents (Edgar et al.*,* 2020). NMDID also contains demographic data for a subset of individuals collected from investigative reports and NOK. This unique dataset permits us to compare medical examiner reports of race and ethnicity to NOK reports, and to explore the association between differences in the two sources and manner and cause of death, for a large, demographically representative sample of the state.

Finally, the history of the US Southwest is distinctive compared to other regions of the US, and New Mexico in particular has a distinctive history of contact between people of Native American, Spanish and non-Spanish or “Anglo” decent over the past 450 years. As a result of this history, today New Mexico has one of the largest proportions of residents in the country who identify on the US Census as being of Hispanic, Latino or Spanish origin (47.7%, US Census, 2020). In contrast to other regions with large Hispanic/Latino populations, many New Mexicans emphasize their Spanish heritage and view themselves as ethnically distinct from more recent immigrants from Latin America. This emphasis on Spanish heritage is in part the result of a history of oppression by Anglo immigrants beginning in the 19th century. Previous studies show misclassification of Hispanic-origin identity on death certificates is particularly pronounced for those who further identified as “Other,” as opposed to Mexican, Puerto Rican, or Cuban [5,11]. “Other” is an especially common choice on the US Census among New Mexican Hispanics [21]. For these reasons, New Mexico can help us understand the importance of regional influences in shaping errors in the assignment of race, ethnicity, manner of death, and cause of death in medicolegal systems throughout the US.

## 2 Materials and methods

### 2.1 Sample

The NMDID contains whole-body CT scans of over 15,000 New Mexico decedents [22]. These individuals died between 2010 and 2017. In addition to the CT scans, descriptive metadata available includes up to 69 variables related to the decedent's demography, life, and death. This information is derived from the death investigation, autopsy records, and for a subset, telephone interviews with NOK. For these telephone interviews, we attempted to contact NOK for 8714 decedents, we were successful in contacting NOK for 2586 decedents, and we gathered data from NOK for 1872 decedents, for a response rate of 21.5%. For our analyses, we only included the subset of these 1872 decedents, 11 and older, for whom both death investigators and NOK described race and ethnicity, and for whom only one race was selected by NOK (n = 1811).

### 2.2 Race and ethnicity

We compared two reports of race and ethnicity, one made by OMI death investigators and one by NOK. All race and ethnicity descriptions at the OMI are made by death investigators, who either work out of the central office in Bernalillo County, the state's population center, or are based in more rural counties. Following similar studies, NOK descriptions were considered accurate due to their personal relationship with the decedent (Edgar et al.*,* 2020).

Slightly different options were available to investigators and NOK for describing race and ethnicity (Table 1). Data used in this study derived from two different pre-existing resources, each following a norm for data collection on race and ethnicity. Death investigators were limited to race categories available on the US Census and New Mexico death certificates. Further, they only had two choices available to describe ethnicity, “Hispanic,” noted by marking a check box in a database, and the default of “not Hispanic,” noted by not checking the box. New Mexicans of Spanish-speaking descent rarely use the term “Latino” and often view “Hispanic” as a descriptor of race as much as, if not more than, ethnicity (Hunley et al.*,* 2018; [23]). When prevented from using Hispanic/Latino for race on the US Census, New Mexicans most often list their race as white. Descriptions from NOK tend to follow these cultural norms. This fact potentially creates mismatches between death investigators, who were required by federal and state standards to describe Hispanic/Latino as an ethnicity, and NOK, who were allowed to answer “Hispanic/Latino” regarding race, ethnicity, or both race and ethnicity in our phone interviews. To account for this region-specific issue, in this study, if a death investigator described a decedent's race as white and their ethnicity as Hispanic/Latino, and NOK used Hispanic/Latino for race, ethnicity (with either white or no description of race), or both race and ethnicity, this was considered an agreement between the investigator and NOK. Table 2 provides example cases of agreement and disagreement between investigators and NOK.

**Table 1:** Race and ethnicity descriptors available to investigator and NOK and their respective samples sizes in the current study.

	Race	n	Ethnicity	n
**Investigators**	1. Asian Indian	1	1. Hispanic/Latino	475
	2. Black or African American	40	2. Not Hispanic/Latino	1330
	3. Japanese	0	3. Blank	0
	4. Korean	0		
	5. Native American	113		
	6. Other Asian	3		
	7. Unknown	0		
	8. Vietnamese	2		
	9. White	1640		
	10. Blank	0		
**Next of Kin**	1. Asian Indian	0	1. Hispanic/Latino	544
	2. Black or African American	51	2. Middle Eastern	3
	3. Chinese	1	3. Not Hispanic/Latino	1179
	4. Filipino	0	4. Unknown	7
	5. Guamanian or Chamorro	0	5. Blank	0
	6. Hispanic/Latino	473		
	7. Japanese	0		
	8. Korean	3		
	9. Native American	129		
	10. Native Hawaiian	0		
	11. Other	4		
	12. Other Asian	2		
	13. Other Pacific Islander	0		
	14. Samoan	0		
	15. Unknown	0		
	16. Vietnamese	2		
	17. White	1143		
	18. Blank	0

**Table 2:** Agreements vs disagreements based on investigators and next of kin responses for race and ethnicity. This list is not all inclusive of the race and ethnicity combinations for investigators.

Examples of Race and Ethnicity Agreement Status				
Race by Investigator	Ethnicity by Investigator	Race by Next of Kin	Ethnicity by Next of Kin	Agreement (Y/N)
Black/African-American	Not Hispanic/Latino	Hispanic/Latino		N
Black/African-American	Not Hispanic/Latino	Black/African-American	Hispanic/Latino	N
Native American	Not Hispanic/Latino	Hispanic/Latino	Hispanic/Latino	N
Native American	Not Hispanic/Latino	Native American	Hispanic/Latin	N
White	Not Hispanic/Latino	Hispanic/Latino		N
White	Not Hispanic/Latino	White	Hispanic/Latino	N
White	Not Hispanic/Latino	Black/African-American	Hispanic/Latino	N
White	Not Hispanic/Latino	Native American		N
White	Not Hispanic/Latino	Black/African-American		N
White	Hispanic/Latino	White	Not Hispanic/Latino	N
White	Not Hispanic/Latino	Hispanic/Latino		N
White	Not Hispanic/Latino	Hispanic/Latino	Hispanic/Latino	N
White	Not Hispanic/Latino	Native American		N
White	Not Hispanic/Latino	Korean	Not Hispanic/Latino	N
White	Not Hispanic/Latino	White	Middle Eastern	N
Black/African-American	Hispanic/Latino	Black/African-American	Hispanic/Latino	Y
Native American	Not Hispanic/Latino	Native American	Unknown	Y
Other Asian	Not Hispanic/Latino	Korean	Not Hispanic/Latino	Y
Other Asian	Not Hispanic/Latino	Other Asian	Unknown	Y
Unknown		White	Not Hispanic/Latino	Y
Unknown	Not Hispanic/Latino	Native American		Y
Unknown		White	Not Hispanic/Latino	Y
White	Hispanic/Latino	Hispanic/Latino	Hispanic/Latino	Y
White	Hispanic/Latino	Hispanic/Latino		Y
White	Hispanic/Latino	Hispanic/Latino	Unknown	Y
White	Hispanic/Latino	Other	Hispanic/Latino	Y
White	Not Hispanic/Latino	White	Unknown	Y

### 2.3 Age and sex

Age and sex data come from the medical investigator records. This information was collected from physical examination, driver's licenses, and other personal documents collected from the decedent. This sample includes only individuals at least 11 years old (n = 1811).

### 2.4 Manner of death and cause of death

At the OMI, cause and manner of death are determined by forensic pathologists. There are five manner of death categories that are standard: accident, homicide, natural, suicide, and undetermined. For cause of death, numerous options are available, and there can be multiple causes listed based on underlying factors. It is the relative contribution of the various causes of death and circumstances surrounding death that allow a medical examiner or forensic pathologist to assign a manner of death. Table 3 provides a list of causes of death seen in this sample in relation to the five respective manners of death categories.

**Table 3:** Manners of death and associated examples of cause of death classifications [24,25].

Manner of Death	Associated Causes of Death
Accident	Substance intoxication, multiple injuries, ethanol intoxication, exposure, drowning, choking, thermal injuries, asphyxia
Homicide	Gunshot wound, multiple injuries, stab wound, asphyxia
Natural	Heart disease, cancer, chronic respiratory disease, stroke
Suicide	Gunshot wound, hanging, substance intoxication, multiple injuries, stab wound
Undetermined	Cannot determine after autopsy, investigation, and laboratory testing

The OMI uses a standardized list of 83 causes of death, 58 of which were seen among the 1811 decedents in this study. For NMDID, the 83 causes of death were grouped with related causes to create 45 summary cause of death codes, e.g., natural, cardiovascular, respiratory disorders, infectious diseases, and bleeding [26]. Of the 45 possible summary codes, 33 were seen in this sample (Table 4).

**Table 4:** List of the 33 causes of death in this sample summarized from the 58 causes of death.

Cause of Death Summary	
Allergy	Infectious Disease
Bleeding	Injuries
Blood Disorders	Malnutrition
Burns	Medical Treatment
Cancer	Natural
Cardiovascular	Neurological
Cerebrovascular	Obesity
Certification	Organ Failure
Choking	Poisoning
Congenital Defect	Respiratory Disorders
Dehydration	Sepsis
Diabetes	Skeletal
Digestive System	Substance
Drowning	Undetermined
Epilepsy	Unnatural
Exposure	Violence
History Of Illness or Injury	

### 2.5 Statistical analyses

For each decedent, we determined whether there was an agreement or disagreement between the investigator and NOK about race and ethnicity. We then calculated frequencies of agreement for each race and ethnicity category. We used logistic regression to determine whether sex or age were predictors of agreement.

We used the Cochran-Mantel-Haenszel (CMH) Statistic to examine the relative risk of a description of a specific ethnicity (regardless of race) or race (regardless of ethnicity) by either the investigator or NOK for all manners of death combined, and for each manner of death separately [27]. We also used the CMH Statistic to examine the relative risk of a description of a specific race or ethnicity by either the investigator or NOK for each cause of death group. In this examination, risk describes the incidence of being identified as a certain race or ethnicity based on manner or cause of death.

We ran several additional tests on subsets of the full sample to account for the unique ethnic landscape in New Mexico (see above). As noted, Hispanic/Latino New Mexicans generally do not distinguish between race and ethnicity with regard to the use of the term, “Hispanic.” The most common distinction New Mexicans make is between Hispanic/Latino and non-Hispanic white. For each manner and cause of death, we examined the risk of the investigator vs. NOK describing a decedent as Hispanic/Latino for the subset of the decedents where NOK described ethnicity as Hispanic/Latino and race as either white or Hispanic/Latino (n = 1608). To explore combinations of Hispanic/Latino ethnicity and races less common in New Mexico, we also performed the CMH tests for subsets of decedents where: 1) NOK described ethnicity as Hispanic/Latino and race as Native American (n = 129); and 2) NOK described ethnicity as Hispanic/Latino and race as Black (n = 51). All statistical analyses were computed using SAS Software v 9.4.

## 3 Results

### 3.1 Agreement about Hispanic/Latino ethnicity

In cases where NOK used Hispanic/Latino for decedent race *or* ethnicity regardless of race, death investigators agreed with NOK 74.38% of the time. This means that in 25.62% of cases, investigators incorrectly described the decedent as non-Hispanic/Latino when the NOK reported the individual as Hispanic/Latino. Conversely, in cases where NOK did not describe the decedent as Hispanic/Latino, investigators incorrectly described the decedent as Hispanic/Latino 1.95% of the time. Overall, across all races and ethnicities, investigators correctly identified decedents 90% of the time. For Hispanic/Latino ethnicity specifically, investigators describe a person's status as Hispanic/Latino or non-Hispanic/Latino correctly 74.38% of the time, creating an overall underestimation of Hispanic/Latino individuals. Table 1 provides the numbers of decedents described by investigators and NOK for each race and ethnicity term.

### 3.2 Agreement about race

We examined investigator/NOK agreement about decedents' race, regardless of ethnicity. For cases where NOK use the term white to describe the decedents race, death investigators agreed 99.4% of the time. Because investigators cannot describe a decedent's race as Hispanic/Latino, but NOK can, agreement cannot be calculated. When NOK described the decedent as Black, the investigators agreed 86.3% of the time. In cases where NOK described the decedent as Native American, the investigators agreed 79.3% of the time.

### 3.3 Age and sex

**Fig. 1:**
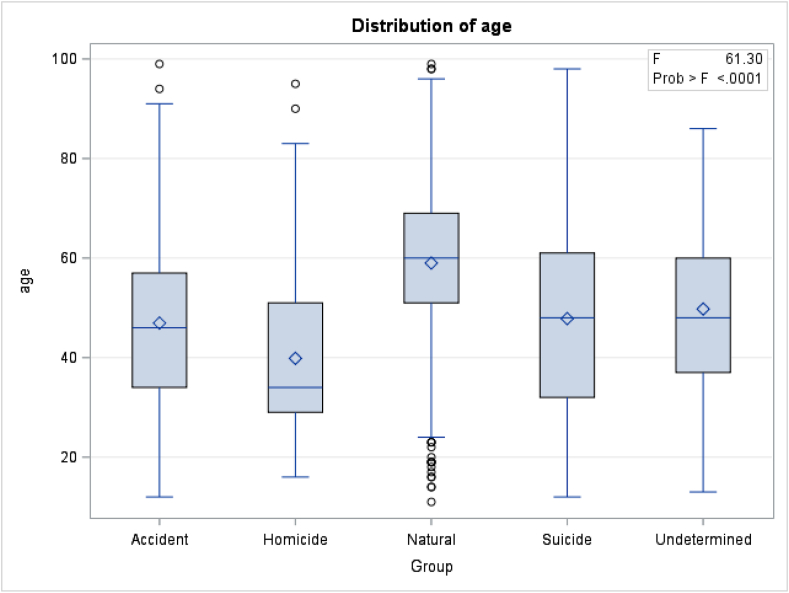
General liner model of age distribution based on manner of death.

The logistic regression included 1580 agreements and 226 disagreements between investigator and NOK. Sex was not a significant predictor of disagreement, but age was (p < 0.001). As decedent age at death increased, the odds of agreement between NOK and the death investigator increased (1.017, 95% CI 1.009–1.025). This means that for each year of life, the odds of agreement about race and ethnicity between investigators and NOK increases by 1.7%. Additionally, there is a significant relationship between age at death and manner of death (p < 0.001); older individuals are more likely to die of natural causes while younger individuals are more likely to die of homicide (Fig. 1).

### 3.4 Manner of death

**Fig. 2:**
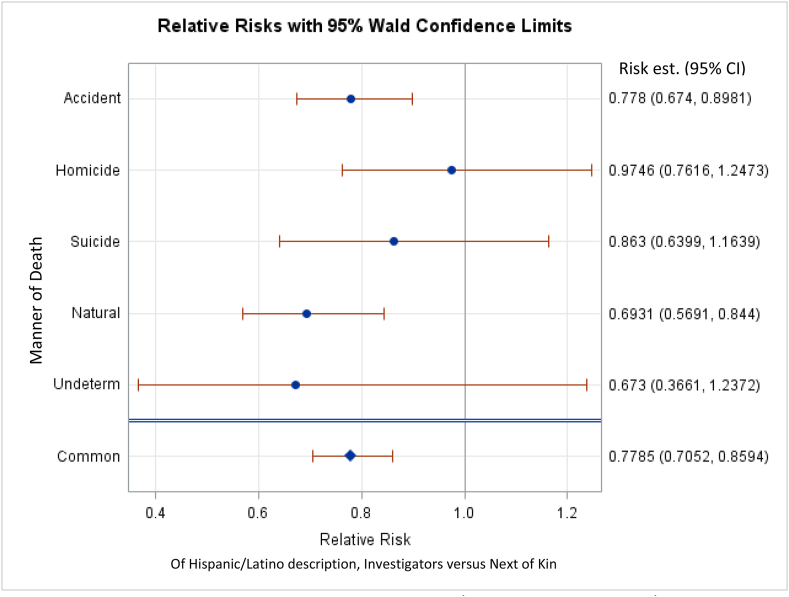
CMH analysis of ethnicity of either Hispanic/Latino or non-Hispanic/Latino for all race descriptions. Risk values less than 1 indicate that the investigator is less likely than NOK to describe a decedent as Hispanic/Latino. Significant results are those for which 95% CIs do not include 1. “Common” refers to all manners of death summed.

For each manner of death, we first examined the relative risk that the investigator vs NOK described a decedent as Hispanic/Latino, regardless of decedent race (Fig. 2). Investigators were significantly less likely than NOK to describe a decedent as Hispanic/Latino, regardless of the manner of death (22.2%; 95%CI: 29.5%, 14.1%). For individual manners of death, investigators were significantly less likely to describe a decedent as Hispanic/Latino when the manner of death was either natural (30.69%, 95%CI: 44.1%, 16.6%) or accidental (22.2%, 95% CI: 33.6%, 11.2%). Results for other manners of death were not statistically significant.

**Fig. 3:**
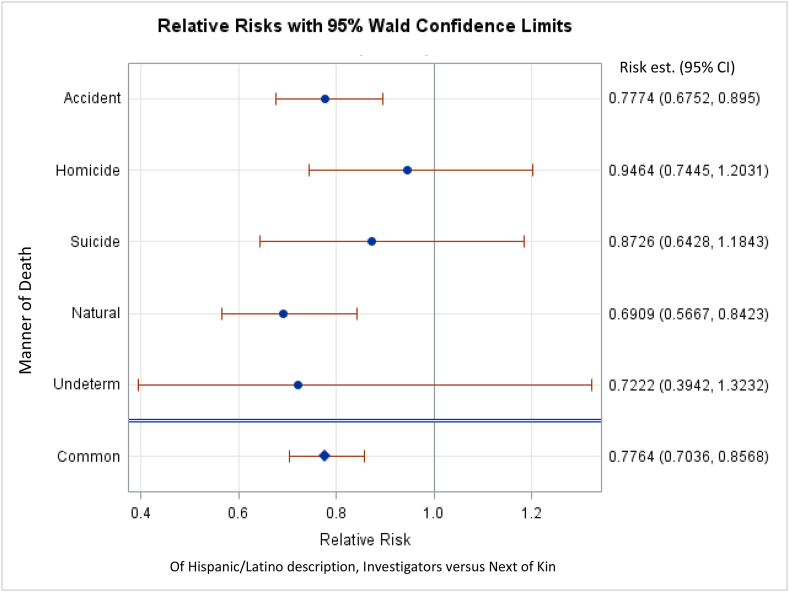
CMH analysis of an ethnicity of either Hispanic/Latino or non-Hispanic/Latino when race was described as White. Risk values less than 1 indicate that the investigator is less likely than NOK to describe a decedent as Hispanic/Latino. Significant results are those for which 95% CIs do not include 1. “Common” refers to all manners of death summed.

We next examined the risk that the investigator vs NOK described a decedent as Hispanic/Latino in the subset of cases for which NOK described the race as either white or Hispanic/Latino (Fig. 3). In this analysis, investigators were 22.36% less likely than NOK to describe decedents as Hispanic/Latino (95% CI:29.7%, 14.4%), regardless of the manner of death. Natural and accidental deaths drive this result. For decedents with natural deaths, investigators were 30.91% less likely (95% CI: 44.4%, 15.8%) to describe a decedent as Hispanic/Latino. For accidental deaths, investigators were 22.26% less likely to describe a decedent as Hispanic/Latino (95% CI: 33.5%, 11.5%). Homicide, suicide, and undetermined manners of death were not significant. There was no manner of death where investigators were more likely to describe a decedent as Hispanic/Latino.

We then tested the relative risk of investigator vs NOK describing a decedent as Hispanic/Latino in only those cases where NOK described the decedent as either Native American or Black. These results were not significant.

**Fig. 4:**
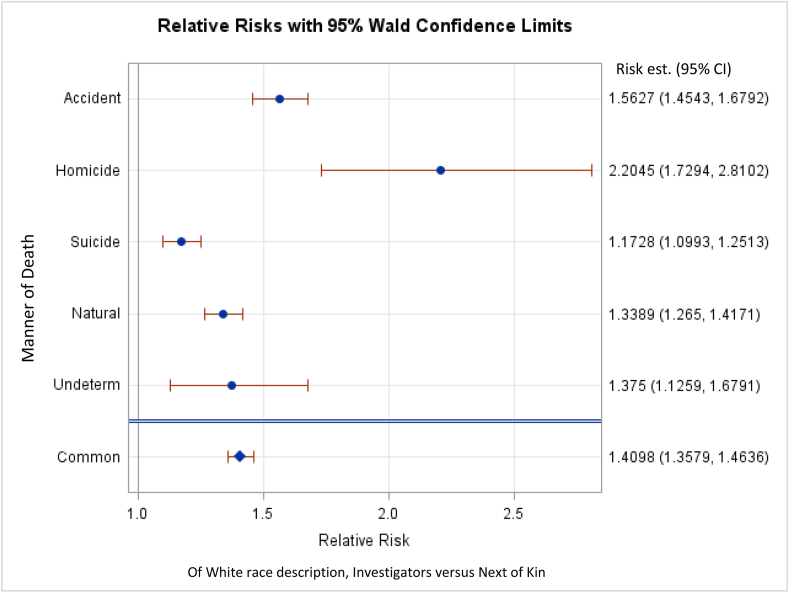
CMH analysis of race (White or any other race) regardless of ethnicity. Risk values are less than 1 indicate that the investigator is more likely than NOK to describe a decedent as White. Significant results are those for which 95% Cis do not include 1. “Common” refers to all manners of death summed.

To examine errors in descriptions of race rather than ethnicity, we next examined the risk that the investigator vs NOK described a decedent as white, regardless of ethnicity (Fig. 4). Overall, investigators were 140.98% as likely to describe decedents as white (95% CI:135.79%, 146.36%), regardless of the manner of death. Results are also significant for each of the five manners of death. The results concerning homicide stand out; investigators were 220.45% as likely to describe a decedent as white than another race when the manner of death was a homicide (95% CI:172.94%, 281.02%).

We also examined errors in the descriptions of race for Native American and Black decedents, regardless of ethnicity. No results were significant (Table 5).

**Table 5:** Relative risk of being identified as Black/African American or Native American by manner of death. No results were significant due to small sample size.

Manner of Death	Black (95% CI)	Native American (95% CI)
**Accident**	1.07 (0.52, 2.2)	0.8814 (0.62, 1.26)
**Homicide**	1.00 (0.33, 3.00)	0.69 (0.31, 1.55)
**Suicide**	0.8 (.22, 2.95)	0.85 (0.45, 1.59)
**Natural**	0.875 (0.49, 1.56)	0.9375 (0.58, 1.52)
**Undetermined**	0.5 (0.05, 5.37)	1.00 (0.30,3.28)
**Overall**	0.9216 (0.62, 1.36)	0.876 (0.69, 1.12)

### 3.5 Cause of death

Table 6 provides relative risks of investigator vs. NOK describing an individual as Hispanic/Latino for each cause of death seen in the sample. Investigators were 22.2% less likely (95% CI: 29.5%, 14.1%) to describe a decedent as Hispanic/Latino regardless of the cause of death code. Only substance abuse and injuries were significant. Substance use is notable, with investigators 24.24% less likely than NOK to describe a decedent as Hispanic/Latino (95% CI:37%, 8.93%). Additionally, investigators were 20.4% less likely to describe the decedent as Hispanic/Latino than NOK if they died from injuries (95% CI:36.55%, 0.22%).

**Table 6:** CMH analysis of 33 summary causes of death for all race and ethnicities. Deaths due to substance abuse and injuries are significant (bolded; α = 0.05) since the confident intervals do not include 1.

COD category	Relative risk of investigator describing as Hispanic/Latino	95% CI
Allergy	*	
Bleeding	0.7143	0.3573, 1.4280
Blood Disorders	*	
Burns	0.6667	0.2441, 1.8209
Cancer	0.7159	0.2989, 1.7147
Cardiovascular disease	0.7785	0.5798, 1.0452
Cerebrovascular	0.7143	0.2912, 1.7519
Certification	*	
Choking	0.9	0.4155, 1.9499
Congenital Defects	*	
Dehydration	1	0.1409, 7.0991
Diabetes	0.6071	0.2186, 1.6859
Digestive System	*	
Drowning	0.8333	0.3059, 2.2700
Epilepsy	0.5	0.1141, 2.1907
Exposure	0.8	0.2442, 2.6213
History of Illness or Injury	*	
Infectious disease	1	0.1409, 7.0991
**Injuries**	**0.7957**	**0.6345, 0.9978**
Malnutrition	*	
Medical error	0.6667	0.2150, 2.0670
Natural	0.669	0.4039, 1.1082
Neurological	1	0.1908, 5.2408
Obesity	0.8	0.3330, 1.9220
Organ failure	1	0.4359, 2.2941
Poisoning	0.5	0.0640, 3.9059
Respiratory disease	0.5556	0.2788, 1.1072
Sepsis	0.7	0.3261, 1.5028
Skeletal	0.6	0.2011, 1.7903
**Substance usage**	**0.7575**	**0.6300, 0.9107**
Undetermined	0.5769	0.1538, 2.1637
Unnatural	*	
Violence	0.91	0.7166, 1.1555

* Indicates statistics were not calculated as sample size was too small.

## 4 Discussion

### 4.1 Race and ethnicity classifications

The OMI 2020 Annual Report states that 32.3% of cases were described as Hispanic/Latino (Office of the Medical Investigator, 2020). This figure contrasts sharply with results of the 2020 Census [28]. In this study, we found that death investigators often incorrectly identify Hispanic/Latino decedents as non-Hispanic. Even though we found that decedent sex did not predict disagreement for Hispanic/Latino identity, it is possible that decedent surname contributed to misidentification for Hispanic/Latino women married to men with non-Hispanic/Latino surnames and for any children who identified as Hispanic/Latino [29]. Other decedent attributes, such as where they were found, clothing, phenotypic attributes, or investigator interactions with NOK, may similarly come into play. Investigators are community members who have chosen to perform difficult work in service to their community. They bring their personal, family, community, and media-moderated experiences to their work. While these experiences make them experts in their own community, they may also introduce cognitive bias into the decisions they make about how to describe decedents.

A related potential cause for misidentifications may be that when investigators are conservative about their classification of race and ethnicity, white is a default response [30,31]. Investigators were significantly more likely to describe decedents as white non-Hispanic for all manners of death, a possible reflection of this default response, despite the fact that Hispanic/Latinos (as they are described by the US Census) make up nearly half the state's population, a greater proportion than white non-Hispanic (36%; US Census, 2020).

### 4.2 Age

The relationship between age at death and manner of death is expected. With age, individuals tend to die more frequently from natural manners of death. Younger individuals have higher instances of homicide, following trends described by the US Department of Justice. In their report, the number of individuals who died by homicide by age peaked between the ages of 25–34 years old between 1980 and 2008. Additionally, in 2008, young adults between the ages 18–24 years old had the highest homicide victimization rate (Cooper and Smith, 2011). The relationship between age at death and manner of death may explain the relationship between age at death and agreement between investigators and NOK about race and ethnicity, as more individuals are described as white when manner of death is natural.

### 4.3 Manner and cause of death classifications

Of the five manners of death, investigators are least likely to classify an individual as Hispanic/Latino, regardless of race, for natural manners of death, followed by accidental deaths. The misrepresentation of race and ethnicity groups in reports of manner of death generates a public misconception of violence within communities [8]. The overestimation of homicide victims as white non-Hispanic may increase fear in that group [32]. Conversely, the artificial inflation of the rate of homicide victims in one group decreases the perceived rate of homicide in other groups. This leads to an underreporting of Hispanic/Latino homicide victims, causing an underappreciation of violence in this community.

Previous findings have shown that there are inconsistencies in the ways race and ethnicity are reported on death certificates when compared to NOK classifications, with the cause of death affecting the classification of race [14]. We found a similar misreporting of Hispanic/Latino decedents when examining cause of death codes. In this sample, when deaths are due to substance abuse or injury, investigators are less likely to describe the decedent as Hispanic/Latino. Both substance abuse and injury cause of death relate to an accidental manner of death; these two causes drive the significant results for the manner of death category. It seems that individuals who die of substance abuse or injury are not as likely to be perceived as Hispanic/Latino by investigators. These results are surprising for two reasons. First, investigators describe race and ethnicity independently from pathologists, who determine cause and manner of death, so there should be no interaction between these factors. Of course, investigators collect data at death scenes, so they may infer potential causes and manners, which might in turn influence their descriptions of decedents. Second, statewide trends suggest that a scene of a drug overdose might influence investigators to describe a decedent as Hispanic/Latino. A state of New Mexico investigation found that between the years 1994 and 2003, 54% of drug related deaths were Hispanic/Latino males. Further, this report found that most of these deaths were due to illicit drugs (75%; [25]. A more recent report highlighted similar results, showing that Hispanic/Latino males had the highest rate of drug overdose deaths in the years 2010–2014 [33]. Despite these statistics, investigators miss Hispanic/Latino ethnicity when describing these decedents.

### 4.4 Potential impacts of cognitive bias

In an analysis of death certificates from Nevada, for children under the age of 6, the manner of death was more likely to be ruled a homicide, rather than an accident, when the child was Black. Additionally, pathologists determined different manners of death depending on information such as race, ethnicity, and the caregiver who was with them [34]. This type of bias also has been shown in an examination of whether cause of death determinations influence how race is reported on death certificates [14]. These authors found that those who died of liver disease were more likely to be classified as Native American, and those who died of homicide were more likely to be classified as Black. There are indications of similar cognitive bias related to the assignment of race and ethnicity in the present study.

The forensic investigation process pools the knowledge of various experts as dictated by the type and complexity of the case. As more people are involved, more evidence is discovered, and experts are exposed to information irrelevant to their portion of the investigation. This sort of trickle-down bias or “bias cascade” builds and may influence any following reports, including medical examiner reports, death certificates, and vital records [35]. The effect of this bias on decedent samples may disadvantage living marginalized groups. Underrepresentation of minority groups in medical examiner data or death certificates, such as seen in this study, obscures health and social inequities experienced by the living populations. There are at least two potential consequences of this underrepresentation. First, public health funding may be misallocated. Second, misrepresentation of the causes of mortality may recapitulate bias within the medicolegal setting.

### 4.5 Race and ethnicity descriptions in forensic casework

Whose description of a decedent's race and ethnicity is accurate? We argue that accuracy is not possible regarding descriptions of race. Since there is no definition of race that reflects a biological reality [36], there is no “right answer” about someone's race. Each person has an idea about their race; this idea can fluctuate and be multileveled. Perhaps more relevant is how members of a decedent's community would describe them [37]. This race description factors into opportunities and resources available during life, shaping structural vulnerability. The most important race description for a decedent may come from NOK. This is especially true for missing persons' cases since NOK and are most likely to provide a description to law enforcement and advocate for investigation. NOK descriptions of race also may relate to how a decedent would have described themselves on the Census, medical forms, and in other demographic data. These are the sources of information we use to understand health disparities.

Using Weber's 1978 definition of ethnicity, a belief in “common descent because of similarities of physical type or of custom or both, or because of memories of colonization and migration,” there can be a reality to ethnicity when people take part in cultural practices that relate to their ethnic background. However, like race, association with an ethnicity is subjective [38]. In New Mexico, the Census category “Hispanic/Latino” is commonly considered equivalent to a race, so the caveats described above apply to descriptions of ethnicity as well. Anthropological research into local community considerations of race and ethnicity should guide data collection on these demographic factors in the medicolegal setting [39].

### 4.6 A structural vulnerability profile in forensic casework

Death investigators, forensic pathologists, and forensic anthropologists all have the goal of identifying and describing a decedent and understanding the cause and manner of death. Each aspect of the death investigation process requires specific input from different experts, and there is constant work being done to improve forensic science by developing better protocols and methods. Within forensic anthropology, the debate about population affinity in forensic anthropological casework is an ongoing exchange, with strong opinions on both sides [40,41]. Conversations about race and ethnicity as a social construct are at the forefront in forensic anthropology but this concept is less discussed in other areas of the medicolegal system. Because race and ethnicity are components of identity, are recorded on death certificates, and will continue to be part of the investigative process, the nuances of collecting and reporting race and ethnicity data are valuable considerations for all involved in death investigations. Our ability to understand disparities in health and mortality, the consequences of structural violence, hinge on our ability to understand the populations that experience them. There are many ways that specific death investigations may, in the future, benefit from the addition of an SVP to forensic anthropological analysis, especially regarding individual identification. Relating evidence of developmental insults, lifelong stress, and early mortality to descriptions of race and ethnicity at the population level can improve our ability to address disparities.

### 4.7 Conclusion

The misidentification of race and ethnicity is not just a problem in New Mexico. Similar errors have been documented across the county [11,14,42]. We show here that disagreement between death investigators and NOK on race and ethnicity descriptions of decedents occurs in one office 25% of the time, with an overall result of underestimating Hispanic/Latino decedents. These findings were found to be associated with some specific manners of death and causes of death. Investigators are less likely to describe a decedent as Hispanic/Latino for accidental and natural deaths, specifically deaths due to substance abuse or injury, and more likely to describe a decedent as White in homicides. The discrepancies found in investigator and NOK reports of race and ethnicity may be attributable to a cognitive bias that influenced how the decedent is perceived by the investigator, or to a general culture of seeing white as a default race descriptor. While death investigators determine neither manner nor cause of death, visual and physical information from a scene, related to manner and cause of death, may affect investigators reports of race and ethnicity. The undercounting of Hispanic/Latino decedents results in casework that does not reflect the demography of the state, misrepresents of the apportionment of accidental and violent causes of death, and creates an incomplete understanding of public health data. Accurate, community specific data, coupled with an SVP, may improve our understanding of health and mortality disparities.

The interconnectivity of descriptions of race and ethnicity and classifications of manner and cause of death emphasizes the need to be aware of the biases that contribute to the errors related to their association. As it relates to the SVP, implicit biases harm communities experiencing social marginalization. The ways in which the SVP is integrated into forensic casework must consider ways to mitigate unwanted prejudice or influence that could ultimately cause further inaccuracies. The errors found is the current study are specifically related to the minority populations that the SVP will be used in improving identification, advancing investigations, and documenting systemic inequities in forensic case reports. Because such errors are higher in marginalized populations, similar errors will carry over into SVP. With this in mind, we hope that as the SVP is further developed, ways to reduce inaccuracies and harm to these communities are prioritized.

## Declaration of competing interest

The authors declare that they have no known competing financial interests or personal relationships that could have appeared to influence the work reported in this paper.
